# Neurological Diseases and Prevalence of Antineuronal Antibodies in Patients with Autoimmune Polyendocrine Syndrome Type 1 – A National Cohort Study

**DOI:** 10.1007/s10875-024-01748-z

**Published:** 2024-06-03

**Authors:** Sini M Laakso, Aino Häkkinen, Outi Mäkitie, Saila Laakso

**Affiliations:** 1 Translational Immunology Research Program, University of Helsinki, Helsinki, Finland; 2 Brain Center, Helsinki University Hospital, Helsinki, Finland; 3 Children’s Hospital and Pediatric Research Center, Helsinki University Hospital, Helsinki, Finland; 4 Research Program for Clinical and Molecular Metabolism, Faculty of Medicine, University of Helsinki, Helsinki, Finland; 5 Folkhälsan Research Center, Helsinki, Finland; 6 Department of Molecular Medicine and Surgery, Karolinska Institutet and Clinical Genetics, Karolinska University Hospital, Stockholm, Sweden

**Keywords:** APS-1, Antineuronal, Neurology, AIRE

## Abstract

Autoimmune polyendocrine syndrome type 1 (APS-1) is a rare monogenic disease caused by mutations in the autoimmune regulator gene. Although the disease-associated autoantibodies mostly target endocrine organs, autoantibodies from patients with APS-1 bind also to rat brain structures. The patients often have GAD65-antibodies, that can cause autoimmune encephalitis. However, neurological manifestations of APS-1 have not been systematically explored. We conducted a retrospective chart review on 44 Finnish patients with APS-1 (median age 38 years, 61% females) and collected all their neurological diagnoses. To assess the prevalence of serum antineuronal antibodies in APS-1, serum samples of 24 patients (median age 36 years, 63% females) were analyzed using a fixed cell-based assay. Of the 44 APS-1 patients, 10 (23%) had also received a diagnosis of a neurological disease. Of these neurological comorbidities, migraine (*n* = 7; 16%), central nervous system infections (*n* = 3; 7%), and epilepsy (*n* = 2; 5%) were the most prevalent. Other diagnoses recorded for single patients were axonal sensorimotor polyneuropathy, essential tremor, idiopathic intracranial hypertension, ischemic stroke, and trigeminal neuralgia. Serum antineuronal antibodies were detected in 42% of patients tested (10/24, 50% females, median age 42 years), GAD65 antibodies being the most common finding. Antibodies against glycine and aquaporin 4 were found in low titers. In four patients, relatively high titers of GAD65 antibodies without coexisting type 1 diabetes were found, but none presented with GAD65-encephalitis. Our study suggests an association between APS-1 and neurological disorders, the mechanisms of which are to be further investigated.

## Introduction

Autoimmune polyendocrine syndrome type 1 (APS-1), also called autoimmune polyendocrinopathy-candidiasis-ectodermal dystrophy (APECED), is a rare monogenic disease caused by mutations in the autoimmune regulator (*AIRE*) gene [1]. The classical triad of disease manifestations includes chronic mucocutaneous candidiasis, hypoparathyroidism, and primary adrenal insufficiency. In addition, type 1 diabetes mellitus (DM1), hypothyroidism, and over 20 other manifestations have been recognized [2]. The disease prevalence is highest in Finland, Sardinia, and among Iranian Jews [1].

AIRE is primarily expressed in medullary thymic epithelial cells, where it promotes the expression of antigens for negative T cell selection [3]. Impaired function of AIRE leads to dysregulation of negative T-cell selection, resulting in self-reactive T-cells escaping into periphery and targeting various organs. Associated autoantibodies are produced and can serve as markers of ensuing autoimmune disease [4]. In mice, Aire expression has been observed in various cell types outside the immune system, including the brain tissue [5]. However, in humans AIRE expression seems to be restricted to immunologically relevant tissues [3, 4].

*AIRE* mRNA was not detected in brain tissue when normal human tissues of non-lymphoid origin were profiled [6]. However, autoantibodies directed against enzymes and cell markers expressed in neurons or glial cells have often been identified in the sera of APS-1 patients, including aromatic L-amino acid decarboxylase (AADC), glutamic acid decarboxylase (GAD65), and tyrosine hydroxylase [4]. Recently, APS-1 patients were reported to produce circulating autoantibodies against acetylserotonin O-methyltransferase (ASMT), a pineal gland enzyme involved in melatonin synthesis, which is expressed solely in the central nervous system [7]. In a study involving 11 patients with APS-1, autoantibodies in sera were able to stain AADC-containing dopaminergic, serotonergic, and noradrenergic neurons in rat brain sections, and sera from six APS-1 patients also stained GABAergic neuronal circuitries. The same staining pattern was observed with cerebrospinal fluid (CSF) available from one of the patients [8]. It is thus possible that autoantibodies and the self-reactive T-cells recognizing the neuronal targets could interfere with neuronal function in patients with APS-1.

Neurological diseases in patients with APS-1 have mostly been described in case reports (Table 1). There is one cohort survey with 158 patients, where posterior reversible encephalopathy syndrome, autoimmune demyelinating disease, polyneuropathy, epilepsy, and ocular myasthenia gravis were reported but the number of patients with each of the diagnoses was not given [9]. Another cohort study including 112 patients reported epilepsy in one patient and cerebellar ataxia in another [2]. A study on 11 patients with rare *AIRE* variants described complex epilepsy and hypothalamic–pituitary dysfunction in one patient [10]. Case reports also include limbic encephalitis with N-methyl-D-aspartate receptor (NMDAR) autoantibodies [11] or autoantibodies against glutamic acid decarboxylase 65-kilodalton isoform (GAD65) [12], cerebellar hypoplasia [13], demyelinating polyneuropathy [14], autoimmune cerebellar degeneration with ataxia and a preceding Miller-Fisher syndrome [15], epilepsy and limb girdle muscular dystrophy [16], and myopathy [17]. In addition, the psychometric profiles of patients with autoimmune polyglandular syndrome are impaired [18]. A link between GAD65 autoantibodies and cerebellar disease has been established [19–21], but much remains to be explored.

**Table 1:** Review of neurological diseases in patients with APS-1 in previous studies

Publication	Neurological symptoms reported	Age at onset and sex	Study method	Population
Garelli et al. 2021 [9]	Posterior reversible encephalopathy syndrome; autoimmune demyelinating disease; polyneuropathy; epilepsy; ocular myasthenia gravis	NA (number of patients with each of the diagnoses also not given)	Cohort survey (*n* = 158)	Italian
Orlova et al. 2017 [2]	Epilepsy; cerebellar ataxia	8y male; 21y male	Cohort study (*n* = 112)	Russian
Podkrajsek et al. 2008 [10]	Complex epilepsy and hypothalamic–pituitary dysfunction in one patient	5y female	Cohort study of 11 patients with rare genetic variants of *AIRE*	Dutch
Kawano et al. 2021 [11]	Limbic encephalitis (anti-NMDAR+)	3-month-old female	Case report	Japanese
Kopczak et al. 2017 [12]	Cerebellar hypoplasia	6y female	Case report	Italian
Chinello et al. 2019 [13]	Limbic encephalitis with epileptic seizures (anti- GAD65+)	21y female	Case report	Dutch
Valenzise et al. 2009 [14]	Demyelinating polyneuropathy	16y and 17y males	Case report	Italian
Berger et al. 2008 [15]	Autoimmune cerebellar degeneration with ataxia and a preceding Miller-Fisher syndrome	24y female (preceding symptom at 9y)	Case report	US
Gazulla Abío et al. 2005 [16]	Epilepsy and limb girdle muscular dystrophy	NA female	Case report	Spanish
Evans et al. 1989 [17]	Myopathy	37y female	Case report	Australian

y years; n number; NA not available; NMDAR N-methyl-D-aspartate receptor; GAD65 glutamic acid decarboxylase 65-kilodalton isoform; *AIRE* autoimmune regulator

We set out to study a large cohort of patients with APS-1 by identifying neurological comorbidities using chart review and exploring the prevalence of autoimmune neuronal antibodies in the serum of these patients.

## Methods

We performed a retrospective chart review on 44 patients with a clinically and genetically confirmed diagnosis of APS-1, as described previously [22], who attended study visits as part of our ongoing studies on the Finnish APS-1 cohort. Seven of the patients were under the age of 18 years. This study was approved by the Ethical committee of Helsinki University Hospital (HUS/1785/2016). All patients participating in the study or their guardians signed an informed consent.

In addition to data on APS-1, we collected all neurological diagnoses (ICD-10 code starting with G) from the patients’ complete medical records available until 2016. The age at symptom onset, investigations performed during diagnostics, severity, and outcome of the neurological disease were assessed. Key features of APS-1 were also reviewed, including autoimmune disease manifestations.

Next, we assessed the prevalence of serum antineuronal antibodies in 24 of these patients, focusing on patients with a neurological diagnosis. Samples were collected 2015–2016. Serum samples were analyzed at the Clinical Immunology Laboratory of Prof Dr Med Stöcker in Gross Grönau, Germany, as subcontracted by Helsinki University Hospital Diagnostic center. The applied method uses fixed cell-based, indirect immunofluorescence assays and BIOCHIP mosaic with brain tissue and recombinant cells to detect neuronal autoantibodies [23]. Specific neuronal antigens tested were: Hu, Ri, ANNA3, Yo, Tr/DNER, myelin, Ma (Ma1, Ma2/Ta), GAD65, amphiphysin, aquaporin-4, NMDA-receptor, AMPA-receptor, GABA-a-receptor, GABA-b-receptor, LGI1, CASPR2, Zic4, DPPX, glycine-receptors, mGluR1, mGluR5, Rho-GTPase activation protein 26, ITPR1, homer 3, MOG, recoverin, neurochondrin, GluRD2, flotillin 1/2, IgLON5, CARPVIII, neurexin-3a, ERC1, Sez6I2, AP3B2, contactin 1, neurofascin 155, neurofascin 186, AT1A3, KCNA2, and dopamine receptor 2.

## Results

### Patients

The study cohort is summarized in Table 2. We studied altogether 44 patients by chart review. Patients included in the study had a wide age range from 7 to 70 years (median 38 years, 61% females), and presented with typical disease manifestations of APS-1 (Table 2). All patients carried biallelic pathogenic *AIRE* variants. Altogether 77% were homozygous for c.769 C > T, p.Arg257Ter, while in the others this variant was compounded with c.967_979del13, (p.Leu323fs), (*n* = 5); c.932G > A, (p.Cys311Tyr), (*n* = 2); or c.891 C > A, (p.Asp297Glu), (*n* = 2).

**Table 2:** Demographics of patients and classical autoimmune disease manifestations of APS-1 in the study cohort

Number of patients	44
Age; median (range)	38 (7–70)
Females; n (%)	27 (61%)
Patients with hypoparathyroidism; n (%)	37 (84%)
Patients with primary adrenal insufficiency; n (%)	36 (82%)
Patients with both hypoparathyroidism and primary adrenal insufficiency; n (%)	30 (68%)
Patients with type 1 diabetes mellitus; n (%)	6 (14%)

### Neurological Diagnoses

Neurological diagnoses recorded are shown in Table 3. Altogether 10 of the 44 patients (23%) had received a neurological diagnosis. All ten patients were homozygous for the AIRE variant c.769 C > T. The most common diagnosis was migraine in seven patients; four of them had also another neurological diagnosis. Epilepsy was diagnosed in two females (5%), of whom one was diagnosed at age 2–5 years with absence epilepsy showing generalized bursts in electroencephalogram (EEG) and brain MRI showed focal cortical dysgenesis. The other subject had an ischemic stroke of unknown etiology at an early age of 40–50, which subsequently caused focal-onset, generalized tonic-clonic seizures. In one patient, at age 25–35 years, polyneuropathy had been verified by electroneuromyography, which indicated axonal sensorimotor subtype. Polyneuropathy was of mild to moderate severity and possibly linked to DM1 which was diagnosed 18 years earlier. One patient had trigeminal neuralgia, where brain imaging identified no vascular compression of the trigeminal ganglion. Idiopathic intracranial hypertension was diagnosed in one patient, and essential tremor in one patient.

**Table 3:** Neurological diagnoses for the APS-1 patients in the cohort (*n* = 44)

Diagnosis	Patient ID	Number of patients (% of all)	Of women (% of patients diagnosed)	Age at diagnosis, median (range) or age range if singular subject
Migraine	#1, #2, #3, #4, #5, #6, #7	7 (15%)	4 (57%)	31 (11–41)
Epilepsy:		2 (5%)	2 (100%)	
-absence epilepsy	#8			2–5
-symptomatic epilepsy	#9			40–50
Axonal sensorimotor polyneuropathy	#4	1 (2%)	0	30–40
Essential tremor	#10	1 (2%)	1 (100%)	10–20
Idiopathic intracranial hypertension	#3	1 (2%)	0	20–30
Ischemic stroke	#9	1 (2%)	1 (100%)	40–50
Trigeminal neuralgia	#6	1 (2%)	0	40–50
Central nervous system infections:		3 (7%)	1 (33%)	
-encephalitis	#6		0	5–10
-meningitis (HSV-2)	#3		0	40–50
-cerebellitis	#7		1	2–5

HSV-2; herpes simplex virus type 2

Central nervous system infections were reported for altogether three subjects (7%); one had encephalitis caused probably by *herpes simplex virus*, one meningitis caused by *herpes simplex virus 2* (HSV-2), and one cerebellitis of unknown cause. The patient with encephalitis presented with decreased level of consciousness, fever, CSF white blood cell count of 185 /cubic mm, and abnormality on electroencephalography that was consistent with encephalitis due to *herpes simplex virus*, but no significant increase of CSF antibodies against *herpes simplex virus* was demonstrated. The patient with HSV-2 meningitis has been reported previously [24]. Both of these patients had autoantibodies against IFNα4 and/or IFNϖ.

#### Antineuronal Autoantibodies

Antineuronal antibodies were analyzed from the serum of 24 APS-1 patients (median age 36 years; 63% females). Eight of them had experienced neurological diseases while 16 had no neurological diagnoses. None of the 24 serum samples showed tissue-specific staining when assessed with rat brain tissue sections. Out of these 24, ten patients (42%) tested positive for at least one antineuronal antibody using recombinant cell assays; 25% (2/8) of patients with neurological disease and 50% (8/16) of those with no neurological diagnoses. The 10 patients (50% females) positive for antineuronal antibodies had a median age of 42 years, six of them (60%) were homozygous for the AIRE variant c.769 C > T, and the median number of APS-1 manifestations was six (range 4–11). Two of those with antineuronal antibodies had presented with neurological comorbidities while the remaining antibody-positive subjects had not been diagnosed with neurological diseases (Table 4). One of the tested patients received immunosuppressive medication and one subcutaneous immunoglobulin G at a substitution therapy dose during sampling, and both of them presented with antineuronal antibodies (Table 4).

**Table 4:** Antineuronal antibodies in the serum of the APS-1 patients in the study cohort (*n* = 22 tested) and neurological diagnoses for these individuals

Antineuronal antibody detected	Titer (for individual patient)	Neurological diagnoses	Number of disease manifestations	Diagnosis of type 1 diabetes mellitus	Age range at serological testing	Time between neurological diagnosis and serological testing (years)
anti-GAD65	1: 3 200	Essential tremor	6*	No	20–30	15
anti-GAD65, anti-glycine	1: 3 200 1: 10	No	6	No	10–20	
anti-GAD65	1: 3 200	No	11**	No	20–30	
anti-GAD65	1: 3 200	No	4	No	40–50	
anti-GAD65	1: 1 000	No	5	No	10–20	
anti-GAD65	1: 1 000	No	7	No	40–50	
anti-GAD65	1: 1 000	No	10	Yes	40–50	
anti-GAD65	1: 100	No	6	Yes	60–70	
anti-GAD65	1: 10	Meningitis, Trigeminal neuralgia	4	No	40–50	6 and 0
anti-aquaporin-4	1: 10	No	6	No	50–60	

*, immunosuppressive medication in use; **, weekly subcutaneous immunoglobulin G substitution therapy in use

Of the antineuronal antibodies, GAD65 was the most common finding, positive for nine patients (9/24, 38%). Only two of the patients positive for GAD65 antibodies had also DM1 (2/9, 22%). There was low positivity for aquaporin-4 antibodies in one patient and for glycine antibodies in one patient who also had GAD65 antibodies. Table 4 shows the exact titers of autoantibodies, as well as the number of APS-1 manifestations and neurological diagnoses for these patients. None of the patients with antineuronal antibodies had been diagnosed with autoimmune encephalitis.

## Discussion

In this national cohort study on 44 subjects with APS-1, we performed a chart review to depict the neurological comorbidities of APS-1 patients. We found a 23% prevalence of any neurological disease, a 7% prevalence of central nervous system infections, and a 5% prevalence of epilepsy. In a subset of 24 patients, we tested for the presence of serum antineuronal antibodies to elucidate the potential role of autoimmunity in the pathogenesis of the neurological comorbidities. Antineuronal antibodies were detected in 42% of the tested APS-1 patients (*n* = 10), and most of them had antibodies against GAD65.

The prevalence and age of onset of the specific neurological diagnoses were overall comparable to the general population, for example for migraine [25], but for epilepsy there was a clear increase compared to the prevalence in the Finnish population, 5% vs. 0.7–1.9% [26, 27]. Importantly, metabolic causes had been carefully excluded for both patients with epilepsy and thus a presentation of DM1, hypoparathyroidism, or adrenal insufficiency was not the cause of repetitive seizures. Epileptic presentations are overrepresented also in our literature review on previous studies (Table 1).

In our study cohort the overall rate of central nervous system infections was 7%. Compared to the recently reported incidence of encephalitis in the Finnish population of 3.1/100,000 person-years [28], we find approximately twenty-fold higher incidence of encephalitis for patients with APS-1. There is a high prevalence of anti-cytokine antibodies, especially anti-IFNα, in patients with APS-1, and the higher level of autoantibodies against IFNα4 associates with more severe herpesvirus infections in APS-1 patients [24]. Autoantibodies neutralizing IFN-α and/or IFN-ω strongly associate with encephalitis caused by the West-Nile virus, whereas asymptomatic cases are seronegative or carry low titers [29]. Whether other viral infections, such as adenovirus, could more easily become symptomatic also in the central nervous system in patients with APS-1 because of these antibodies, remains to be studied further. Measuring levels of anti-cytokine antibodies from the CSF could give more evidence to support such a mechanism.

GAD65-encephalitis presents most commonly as epilepsy, ataxia, or stiff person syndrome [30]. Typically, GAD65-encephalitis gives rise to very high titers of antibodies in the serum, measured in tens of thousands or at least thousands on the scale found also here [31]. For five of the nine patients seropositive for GAD65 in our study, the antibody levels were of low range, compatible with false positive detection or DM1 that had been diagnosed in two of these patients. However, for four patients, the titer was reported in thousands. Interestingly, one of these patients had a diagnosis of essential tremor, in which differential diagnostics for ataxia can be difficult. However, most of the patients with GAD65 autoantibodies had not been diagnosed with a neurological disease, and caution should be elicited in interpreting other than the typical manifestations as GAD65-encephalitis [32].

Altogether 38% of the tested patients presented with autoantibodies against GAD56, which is strikingly high in comparison to 2% in the general populations in the Nordic countries [33]. GAD65 antibodies are thought to rise secondary to T-cell mediated cell lysis both in DM1 and GAD65-encephalitis. In both diseases, the antibodies targeting this intracellular protein bind small peptides of GAD65, further in line with a by-stander effect of the initial process of cellular lysis. In both DM1 and GAD65-encephalitis, CD8 + T cell responses against target organs have been reported, and an association to HLA-A*02:01 has been found [34]. Further research is needed on the pathogenetic mechanisms causing these different disease presentations, which also can manifest in same individuals [35]. Whether GAD65 autoantibodies have a mechanistic role in the clinical manifestations of APS-1 outside of DM1 or its prodromal phase cannot be deducted with certainty from our results. Long-term follow-up studies are needed to determine their mechanistic role in APS-1.

A limitation of our study is the lack of CSF testing for antineuronal autoantibodies. With the findings of our study, this should be addressed in future studies on patients with repetitive seizures and APS-1. Another limitation is the retrospective study setting. Whether detected autoantibodies in asymptomatic patients have clinical significance, will be revealed only after follow-up that is also required for the estimation of life-time risk of neurological diseases associated with APS-1. Also, testing with a live neuron -based assay could have provided further understanding on the role of these autoantibodies, and this should be addressed in the future. We did not include symptomatic diagnoses such as headache or psychiatric diagnoses in the analyses, although disturbed autoimmunity against neuronal structures may be involved in their pathogenesis as well. Neither were we able in our retrospective chart review to rule out or confirm an autoimmune mechanism for etiology of neurological diagnoses such as stroke. None of the patients in our study had been evaluated for the possibility of autoimmune encephalitis at the acute phase when the sensitivity to detect antineuronal antibodies would have been the highest. Despite these limitations, we consider these findings important as this represents the first study to evaluate both neurological manifestations and antineuronal antibodies in a large cohort of patients with APS-1.

To conclude, our thorough review of patient records for neurological comorbidities in patients with APS-1 found a high rate of epilepsy and central nervous system infections but also several other neurological disorders. With the high prevalence of neurological disorders overall and of GAD65-autoantibodies in APS-1 presenting without DM1, the mechanisms of these associations should be addressed in future studies.

## Data Availability

The datasets generated and analysed during the current study are available from the corresponding author on reasonable request.
